# 

**Published:** 1860-09

**Authors:** 


### CONTENTS FOR SEPTEMBER, 1860.

### ORIGINAL COMMUNICATIONS.

Inflammatory Affections of the Female Breasts. By W. H. BYFORD, M. D., of Chicago. 513
Notes on Surgical Cases. By E. ANDREWS, M. D. .... 546
Case of Poisoning by Laudanum.---Cold Water Treatment. By H. WARDNER, M. D.... 548
Paraphlegia Cured by the Use of Magnectic Electricity. By Dr. CERF, of Wheeling, Ill. 550
Clinical Report. By Dr. N. S. DAVIS.... 551

### BOOK AND PAMPHLET NOTICES.

On the Diseases, Injuries and Malformations of the Rectum and Anus. With Remarks on Habitual Constipation. By T. J. ASHTON, M. D.... 558
WALSHE on Diseases of the Lungs.... 561

### SELECTIONS.

New York Medical and Surgical Society.—Discussion on Diphtheria.... 562
Can a Woman Remain Ignorant of her Pregnancy up to the time of her Delivery? .... 570
Transmission of Secondary Syphilis.... 571
Special Hospitals.... 571
Somnambulists Fined.... 572

### EDITORIAL.

Illinois State Medical Society.... 572
Medical Schools in Chicago.... 573
Museum and Library of the Medical Department of the Lind University.... 575

